# Efficacy and Safety of HAIC Combined with PD-(L)1 Inhibitors and Bevacizumab Versus HAIC with PD-(L)1 Inhibitors and TKIs in Advanced Hepatocellular Carcinoma: A Retrospective Cohort Study

**DOI:** 10.3390/cancers18020314

**Published:** 2026-01-20

**Authors:** Zizhuo Wang, Wei Xu, Songlin Song, Yanqiao Ren, Jiacheng Liu, Yiming Liu, Xuefeng Kan, Chuansheng Zheng, Bin Liang

**Affiliations:** 1 Department of Radiology, Union Hospital, Tongji Medical College, Huazhong University of Science and Technology, Jiefang Avenue 1277, Wuhan 430023, China; wzz199902@163.com (Z.W.); m202476500@hust.edu.cn (W.X.); song9413lin@sina.cn (S.S.); 2022xh0059@hust.edu.cn (Y.R.); jiacheng6jc@163.com (J.L.); lymtongji@163.com (Y.L.); xkliulang1314@163.com (X.K.); hqzcsxh@sina.com (C.Z.); 2 Hubei Provincial Clinical Research Center for Precision Radiology & Interventional Medicine, Wuhan 430023, China; 3 Hubei Key Laboratory of Molecular Imaging, Wuhan 430023, China

**Keywords:** bevacizumab, tyrosine kinase inhibitor, programmed death-(ligand)1, hepatic arterial infusion chemotherapy, hepatocellular carcinoma

## Abstract

Background: The combination of hepatic arterial infusion chemotherapy (HAIC) with immune checkpoint inhibitors (ICIs) and anti-angiogenic agents represents a potential therapeutic strategy for advanced hepatocellular carcinoma (HCC). This study aimed to compare the efficacy and safety of triple therapies combining HAIC with ICIs and either bevacizumab or tyrosine kinase inhibitors (TKIs) in these patients. Methods: This retrospective single-center study enrolled 65 consecutive patients with advanced HCC who received HAIC combined with ICIs plus either bevacizumab (bevacizumab group, *n* = 31) or TKIs (TKIs group, *n* = 34) between June 2021 and June 2023. Primary endpoints included progression-free survival (PFS), objective response rate (ORR), duration of response (DOR), and safety profiles. Results: The bevacizumab group demonstrated significantly prolonged median PFS (10.9 vs. 7.4 months, *p* = 0.001) and higher ORR (83.9% vs. 61.8%, *p* = 0.047) compared with the TKIs group. DOR was longer in the bevacizumab group (7.9 vs. 5.3 months, *p* = 0.008). Median overall survival (OS) was not reached in the bevacizumab group versus 22.6 months in the TKIs group. Grade 3–4 adverse events occurred in 67.7% of the bevacizumab group and 73.5% of the TKIs group, with distinct toxicity profiles. Gastrointestinal hemorrhage (45.2%) and gastric ulcer (22.6%) predominated in the bevacizumab group, whereas the TKIs group exhibited more hepatic enzyme elevations (aspartate aminotransferase, 67.6%; alanine aminotransferase, 61.8%), proteinuria (29.4%), diarrhea (26.5%), hand-foot syndrome (20.6%), and reactive cutaneous capillary endothelial proliferation (11.8%). Conclusions: Bevacizumab-containing triplet therapy was associated with improved tumor control and delayed progression compared to TKIs-based regimens in advanced HCC. The higher bleeding risk in the bevacizumab group highlights the necessity of standardized baseline evaluation and adequate preventive measures.

## Simple Summary

This retrospective study compared the efficacy and safety of two triplet regimens—hepatic arterial infusion chemotherapy (HAIC) plus immune checkpoint inhibitors combined with either bevacizumab or tyrosine kinase inhibitors (TKIs)—in patients with advanced hepatocellular carcinoma (HCC). Among 65 enrolled patients, the bevacizumab-based regimen suggested improved tumor control, with a higher objective response rate (83.9% vs. 61.8%) and longer median progression-free survival (10.9 vs. 7.4 months) compared to the TKI-based regimen. Both regimens exhibited manageable toxicity profiles, but with distinct adverse event profiles. These real-world findings suggest that the triple combination containing bevacizumab may offer a more effective treatment option, warranting further validation in prospective clinical trials.

## 1. Introduction

Hepatocellular carcinoma (HCC) ranks as the sixth most prevalent malignancy globally and contributes to the third-highest cancer-related mortality, with China bearing a disproportionately high disease burden [1]. Many patients are diagnosed at an advanced stage, for whom curative treatment options are not indicated [2,3]. While current guidelines recommend combinations of immune checkpoint inhibitors (ICIs) and anti-angiogenic therapy as first-line treatment for advanced HCC, particularly those classified as Barcelona Clinic Liver Cancer (BCLC) stage C [3], the clinical outcomes remain suboptimal [3,4]. Established regimens, including atezolizumab plus bevacizumab and pembrolizumab plus lenvatinib, have median OS that seldom surpasses 24 months, highlighting a pressing need for more effective therapeutic strategies.

Hepatic arterial infusion chemotherapy (HAIC), particularly with the FOLFOX regimen, has emerged as a potent locoregional treatment that enhances systemic therapy efficacy in advanced HCC. By delivering high concentrations of chemotherapy directly to the liver, HAIC achieves potent tumor control with limited systemic exposure [5,6,7,8]. Real-world studies demonstrate that FOLFOX-HAIC combined with anti-angiogenic therapy and ICIs can lead to improved therapeutic efficacy [9,10,11,12], with median progression-free survival (PFS) showing significant enhancements over conventional therapy combining ICIs and anti-angiogenic therapy [13,14,15,16]. These findings position HAIC-based combination therapy as a promising advancement in advanced HCC management.

However, the optimal anti-angiogenic agent to combine with HAIC and ICIs remains unclear. While anti-angiogenic drugs—such as VEGF-targeting monoclonal antibodies (e.g., bevacizumab) and multi-target tyrosine kinase inhibitors (TKIs; e.g., lenvatinib)—form a cornerstone of systemic therapy [17,18], their comparative performance in this triplet setting is not well-established [19,20]. Direct comparisons between bevacizumab and TKIs combined with HAIC and ICIs are lacking. This study therefore aims to compare the efficacy and safety of these two anti-angiogenic strategies in the context of HAIC-ICI combination therapy, to inform the selection of anti-angiogenic agents in clinical practice for advanced HCC.

## 2. Materials and Methods

### 2.1. Study Design and Participants

This retrospective cohort study was conducted in accordance with the Declaration of Helsinki and approved by the Institutional Review Board of Union Hospital, Tongji Medical College, Huazhong University of Science and Technology (Approval No. 2024-0725). Informed consent requirement was waived by the ethics committee in accordance with national regulations for retrospective analyses.

Consecutive patients with HCC treated at our hospital between June 2021 and June 2023 were retrospectively reviewed. Eligible participants received first-line therapy comprising FOLFOX-HAIC, ICIs and anti-angiogenic therapy. Two comparative cohorts were established based on targeted therapy regimens: the bevacizumab cohort (HAIC combined with ICIs and bevacizumab) and the TKIs cohort (HAIC combined with ICIs and TKIs). The selection of specific PD-(L)1 inhibitors and anti-angiogenic agents was not protocol-defined but reflected real-world clinical practice, influenced by contemporary drug availability, reimbursement policies, and clinical judgment.

Inclusion criteria encompassed (1) age ≥ 18 years; (2) HCC diagnosis confirmed histologically or clinically per European Association for the Study of the Liver (EASL) criteria; (3) BCLC stage C classification; (4) Eastern Cooperative Oncology Group (ECOG) performance status 0–1; and (5) completion of ≥2 treatment cycles with the FOLFOX-HAIC/ICI/anti-angiogenic therapy combination. Exclusion criteria included (1) concurrent HCC-directed therapies (e.g., transarterial chemoembolization [TACE] or radiotherapy) during the study period; (2) history of other malignancies; (3) incomplete essential clinical/imaging datasets; and (4) loss to follow-up (>3 months).

### 2.2. FOLFOX-HAIC Procedure

All patients underwent a standardized HAIC procedure performed by two experienced interventional radiologists (Songlin Song and Bin Liang, with >10 years of expertise). Prior to treatment, patients underwent a comprehensive evaluation, including medical history, physical examination, laboratory tests (complete blood count, hepatic and renal function, coagulation profile, and tumor markers), and contrast-enhanced cross-sectional imaging (CT or MRI) [21].

The procedure was performed via femoral artery access using the Seldinger technique. Initial diagnostic angiography of the hepatic vasculature was conducted with a 5-Fr catheter. Subsequently, a 2.7-Fr microcatheter was advanced superselectively into the tumor-feeding artery. When superselective catheterization was not feasible for entire tumor coverage, the catheter tip was positioned in the right or left hepatic artery. Prophylactic embolization of non-target arteries was performed as needed to optimize drug delivery [22,23,24].

All patients received the same modified FOLFOX6 chemotherapy regimen: oxaliplatin (85 mg/m^2^ infused over 2 h), leucovorin (200 mg/m^2^ over 1 h), followed by fluorouracil (400 mg/m^2^ bolus then 2400 mg/m^2^ continuous infusion over 46 h). Treatment cycles were repeated every 3 weeks until disease progression or unacceptable toxicity. Dose adjustments in subsequent cycles were permitted based on predefined criteria for hepatic function and treatment tolerance [25,26]. Any procedure-related complications were documented within the overall assessment of adverse events.

### 2.3. Immunotherapy Protocol

PD-(L)1 inhibitors (sintilimab 200 mg, tislelizumab 200 mg, camrelizumab 200–250 mg, atezolizumab 1200 mg, or triplimab 240 mg) were administered intravenously every 3 weeks. Each infusion was given 3 days after HAIC completion. Treatment was continued until disease progression or the occurrence of grade ≥ 3 treatment-related adverse events. Dose reductions were not permitted; however, treatment could be temporarily interrupted or permanently discontinued for unresolved toxicities, consistent with CTCAE v5.0 guidelines.

### 2.4. Vascular Targeted Therapy Protocol

The anti-angiogenic therapies in this study comprised tyrosine kinase inhibitors (TKIs: apatinib 250 mg, donafenib 200 mg, or lenvatinib 8 mg, all administered orally once daily) and the monoclonal antibody bevacizumab (7.5 mg/kg intravenously every 3 weeks). To mitigate peri-procedural risks, oral TKIs were withheld from 48 h before to 72 h after each HAIC session, while bevacizumab infusion was scheduled for at least 72 h after HAIC. Treatment modifications, including dose reduction, dose interruption, or treatment discontinuation, were implemented to manage treatment-related adverse events, in accordance with institutional guidelines and the approved prescribing information.

### 2.5. Efficacy and Safety Assessment

Baseline and clinical data were retrospectively collected from electronic medical records. Systematic radiological surveillance incorporating contrast-enhanced abdominal CT/MRI and chest CT was conducted at baseline, q6w during the initial 12-month therapeutic phase, followed by q12w intervals until disease progression or mortality. Dual-blinded independent radiographic evaluation was conducted by two board-certified radiologists (Bin Liang and Songlin Song) with >10 years of hepatobiliary imaging experience.

The efficacy evaluation indices included PFS, OS, 1-year OS rate, ORR, disease control rate (DCR), time to response (TTR), and duration of response (DOR). PFS was defined as the interval from treatment initiation to radiologically confirmed progression or all-cause mortality (whichever occurred first). ORR was defined as the proportion of patients with a complete response (CR) or partial response (PR). DCR was defined as the proportion of patients with a CR, PR, or stable disease (SD). TTR was defined as the time from treatment initiation to the first recorded CR or PR for patients with CR or PR. DOR was defined as the time from the first recorded CR or PR to disease progression or death for patients with CR or PR. The assessment of PFS, ORR, DCR, TTR, and DOR was based on RECIST 1.1. OS was defined as the time from treatment initiation to death from any cause.

Safety monitoring encompassed comprehensive documentation of TRAEs during therapeutic cycles and surveillance periods. All AEs were graded according to CTCAE v5.0, with protocol-defined allowances for dose reduction, dose interruption, or treatment discontinuation.

### 2.6. Statistical Analysis

Categorical variables were summarized as frequencies and percentages. Continuous variables, after assessment for normality, are presented as mean ± standard deviation or median (range), as appropriate. Group comparisons were performed using Student’s *t* test, Wilcoxon rank-sum test, Chi-square test, or Fisher’s exact test, depending on the variable type and distribution. Time-to-event endpoints, including PFS, OS, TTR, and DOR, were estimated by the Kaplan–Meier method and compared with the log-rank test. Hazard ratios (HRs) with 95% CIs were calculated using univariate Cox models, with patients censored at their last valid imaging assessment. Variables with *p* ≤ 0.05 in univariate analysis were considered for inclusion in a multivariable Cox proportional hazards model to identify independent prognostic factors. A two-sided *p*-value < 0.05 was considered statistically significant. Statistical analysis was performed using SPSS 27.0 (SPSS, Chicago, IL, USA) and GraphPad Prism 10.0 (GraphPad Software, La Jolla, CA, USA).

## 3. Results

### 3.1. Patient Characteristics

**Figure 1:**
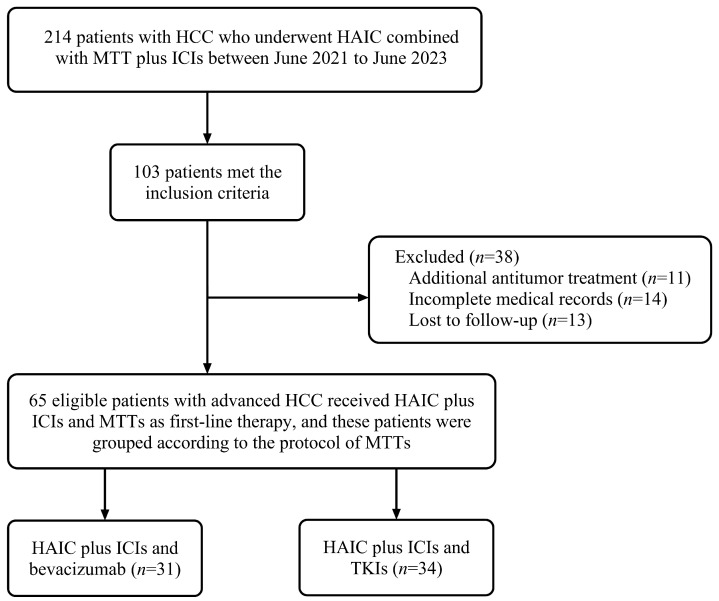
Flowchart of patient selection and study design. From June 2021 to June 2023, 214 patients with HCC who received HAIC combined with anti-angiogenic therapy and ICIs were initially considered. After applying the inclusion and exclusion criteria, 65 eligible patients with advanced HCC were enrolled as first-line therapy. These patients were subsequently divided into two groups based on the protocol of anti-angiogenic therapy: one receiving HAIC plus ICIs and bevacizumab (*n* = 31), and the other receiving HAIC plus ICIs and TKIs (*n* = 34).

Between June 2021 and June 2023, 214 consecutive hepatocellular carcinoma (HCC) patients were screened for HAIC in combination with ICIs and anti-angiogenic therapy. Following eligibility assessment, 65 patients were enrolled, including 31 assigned to HAIC plus ICIs and bevacizumab and 34 to HAIC plus ICIs and TKIs (Figure 1). In the TKIs group, the specific agents used were lenvatinib (*n* = 15, 44.1%), donafenib (*n* = 12, 35.3%), and apatinib (*n* = 7, 20.6%). The study population exhibited advanced disease burden. In the bevacizumab cohort, 67.7% (21/31) had tumors ≥ 10 cm in maximal diameter, 87.1% (27/31) demonstrated portal vein invasion, and 32.3% (10/31) presented with extrahepatic metastases. The corresponding figures in the TKIs group were 76.5%, 79.4%, and 55.9%, respectively. The other baseline characteristics are presented in Table 1. No statistically significant differences were observed between the two groups for all other baseline characteristics (*p* > 0.05).

### 3.2. Treatment Exposure and Compliance

The final follow-up was conducted in May 2025. Overall median follow-up duration was 25.8 months (range: 4.3–40.0), with medians of 23.9 months (range: 6.1–35.3) for the bevacizumab cohort and 25.8 months (range: 4.3–34.1) for the TKIs cohort. All participants underwent HAIC procedures with radiologically confirmed technical success, yielding a 100% technical success rate in both groups. The median HAIC cycles in the bevacizumab and TKIs groups were 6 (range: 3–9) and 5 (range: 2–9) cycles, respectively. The median ICIs cycles in the two groups were 11 (range: 3–33) and 7 (range: 2–33) cycles, respectively. The median number of anti-angiogenic therapy cycles was 11 (range: 3–33) in the bevacizumab group and 6 (range: 1–24) in the TKIs group. Complete therapeutic agent profiles, including regimens of ICIs and anti-angiogenic agents and their cycle distributions, are provided in Tables S1 and S2.

### 3.3. Therapeutic Efficacy

**Figure 2:**
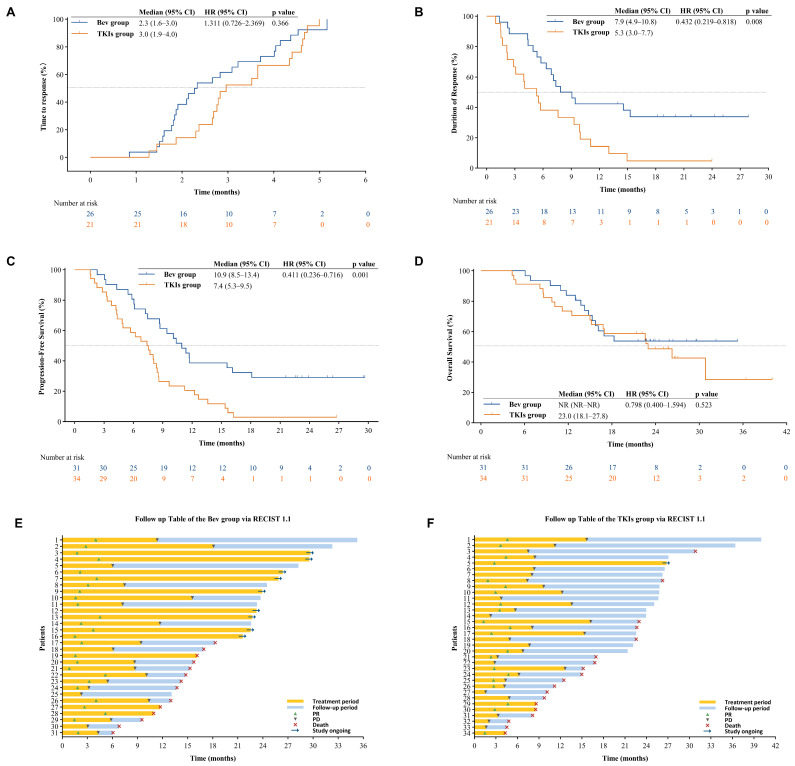
Kaplan–Meier analysis of time to response (**A**), duration of response (**B**), progression-free survival (**C**), and overall survival (**D**). Differences between groups were compared using the log-rank test. A *p*-value of less than 0.05 was considered statistically significant. The univariate Cox proportional hazards model was employed to calculate HRs and their corresponding 95% CIs. Swimmer plot of treatment response and clinical course (**E**,**F**). The swimmer plot illustrates the individual treatment duration, response, and subsequent clinical outcomes for each patient in the two treatment groups, as evaluated by RECIST 1.1. The dashed lines in (**A**–**D**) represent 50% of the Y-axis rate.

Efficacy outcomes between the bevacizumab and TKIs groups are summarized in Table 2. According to RECIST 1.1, the bevacizumab group demonstrated significantly higher ORR (83.9% vs. 61.8%, *p* = 0.047) and DOR (7.9 vs. 5.3 months, *p* = 0.008) compared to the TKIs group (Figure 2A). There were no significant differences between the groups in TTR (2.3 vs. 3.0 months, *p* = 0.366; Figure 2B) or DCR (96.8% vs. 85.3%, *p* = 0.243).

At the end of follow-up, PFS events occurred in 71.0% (22/31) patients of bevacizumab group and 97.1% (33/34) patients of TKIs group, respectively. The PFS was markedly prolonged in the bevacizumab group, with a median PFS of 10.9 months (95% CI: 8.5–13.4) versus 7.4 months (95% CI: 5.3–9.5) in the TKIs group (*p* = 0.001; Figure 2C). At data cut-off, the median OS was not reached in the bevacizumab group (95% CI: NR–NR; 1-year survival rate: 83.9%), compared to 23.0 months (95% CI: 18.1–27.8; 1-year survival rate: 73.5%) in the TKIs group (Figure 2D). The follow-up tables assessed using RECIST 1.1 for both groups are shown in Figure 2E,F.

**Figure 3:**
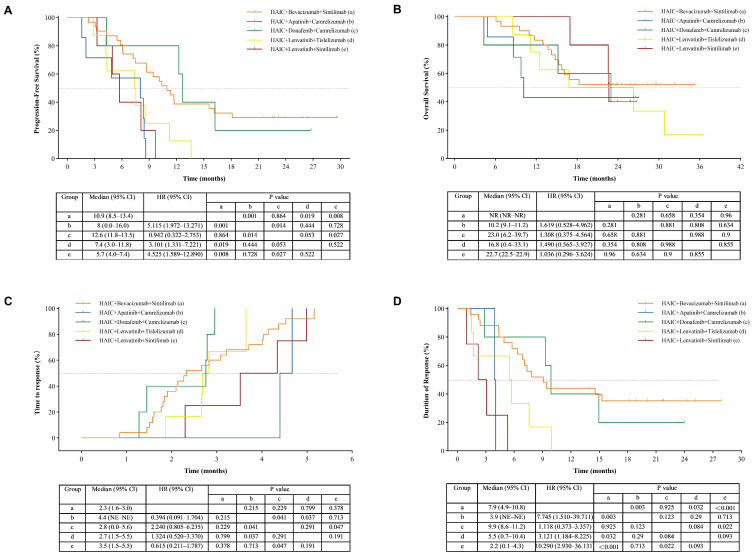
Kaplan–Meier analysis of progression-free survival (**A**), overall survival (**B**), time to response (**C**), and duration of response (**D**) in the subgroup. Differences between groups were compared using the log-rank test. A *p*-value of less than 0.05 was considered statistically significant. The univariate Cox proportional hazards model was employed to calculate HRs and their corresponding 95% CIs.

The multivariable Cox regression model met the proportional hazards assumption. On multivariate analysis, the bevacizumab-containing regimen (HAIC plus ICIs and bevacizumab) remained an independent predictor of prolonged PFS (hazard ratio [HR]: 0.47; 95% CI: 0.26–0.85; *p* = 0.012) when assessed by RECIST 1.1 (Table 3). Subgroup analyses were also performed for specific drug combinations with ≥5 patients each. The HAIC–sintilimab–bevacizumab subgroup showed a potential efficacy trend compared to other subgroups. However, no significant difference was observed when it was directly compared with the HAIC–camrelizumab–donafenib subgroup (Figure 3). It should be noted that the latter subgroup comprised only 5 patients, which limits the statistical power of this direct comparison and precludes definitive conclusions. These exploratory findings warrant validation in larger cohorts. To further explore potential differences among the three individual TKIs within the TKIs group, we conducted a PFS subgroup analysis stratified by the specific TKI agent (lenvatinib, donafenib, and apatinib). No significant difference in PFS was observed among these three TKI subgroups (Figure S1).

### 3.4. Safety Profile

All patients in both cohorts experienced at least one TRAE of any grade (Table S3). No grade 5 adverse events occurred. Hematologic toxicities, hypoalbuminemia, hypertension, abdominal pain, fever, fatigue, and weight loss were commonly observed in both groups. However, differences emerged for specific AEs. Gastrointestinal hemorrhage (45.2% vs. 8.8%, *p* = 0.002) and gastric ulcer (22.6% vs. 2.9%, *p* = 0.040) were significantly more frequent in the bevacizumab cohort. Among the 14 patients with gastrointestinal hemorrhage in the bevacizumab cohort, 13 (92.9%) were grade 1–2 and 1 (7.1%) was grade 3–4. The majority of these cases were detected through routine fecal tests showing blood cells and typically did not require specific intervention. In contrast, patients in the TKIs cohort more frequently experienced elevated transaminase levels (AST: 67.6% vs. 32.3%, *p* = 0.003; ALT: 61.8% vs. 19.4%, *p* = 0.002) and hand-foot syndrome (20.6% vs. 0%, *p* = 0.018).

In the bevacizumab cohort, 29.0% (9/31) of patients required dose interruption, with treatment discontinuation occurring in 3.2% (1/31). No dose reductions were implemented. Conversely, in the TKIs cohort, dose reduction was required in 26.5% (9/34) of patients, while 2.9% (1/34) required dose interruption and 2.9% (1/34) underwent treatment discontinuation. Detailed information on treatment adjustments for both groups is presented in Table S4.

## 4. Discussion

This retrospective study suggests that the combination of HAIC and ICIs with bevacizumab is associated with significantly better key efficacy outcomes—including ORR, PFS, and DOR—compared with TKI-based triple therapy in advanced HCC. Multivariate analysis further identified the bevacizumab-containing regimen as an independent predictor of prolonged PFS.

Specifically, the bevacizumab group achieved a higher ORR (83.9% vs. 61.8%, *p* = 0.047) and a longer median PFS (10.9 vs. 7.4 months, *p* = 0.001). These findings are consistent with most previous studies on HAIC-based triplet therapies, which reported median PFS ranging from 6.6 to 13.8 months [27,28,29,30]. In contrast, He et al. reported superior efficacy for a TKI-based regimen [31], which might be attributable to the inclusion of stage B patients and greater heterogeneity in systemic agents. Additionally, the bevacizumab group showed a significantly longer median DOR (7.9 vs. 5.3 months, *p* = 0.008). Subgroup analysis suggested a potential efficacy advantage for the HAIC–sintilimab–bevacizumab combination, although no significant difference was found in a direct comparison with the HAIC–camrelizumab–donafenib subgroup; these results require cautious interpretation due to the limited sample size. Interestingly, although the early survival signals (6-month OS rate: 100% vs. 91.2%; 12- month OS rate: 83.9% vs. 73.5%) favored the bevacizumab-containing triplet regimen, the 18-month landmark data (57.1% vs. 58.8%) suggest that the long-term overall survival rates between the two groups may become comparable. This underscores that the current data analysis remains at a relatively early stage, and longer follow-up is essential to determine whether any initial survival benefit can be sustained.

Both regimens demonstrated no grade 5 TRAEs and had low rates of permanent treatment discontinuation (bevacizumab: 3.2%; TKIs: 2.9%). The toxicity profiles, however, differed. The bevacizumab group exhibited higher rates of gastrointestinal events, including hemorrhage (45.2% vs. 8.8%; 3.2% grade 3–4) and ulcers (22.6% vs. 2.9%; 9.7% grade 3–4), consistent with its anti-VEGF mechanism. In contrast, the TKIs group showed increased hepatic toxicity (AST elevation: 67.6% vs. 32.3%; ALT elevation: 61.8% vs. 19.4%) and higher incidences of proteinuria (29.4%), diarrhea (26.5%), hand-foot syndrome (20.6%), and RCCEP (11.8%), mirroring established TKIs toxicity patterns.

These distinct profiles required distinct management approaches. For bevacizumab, management primarily involved dose interruption (29.0%) rather than dose reduction, allowing maintenance of therapeutic intensity despite bleeding risks. For TKIs, dose reduction (26.5%) was more commonly required to manage toxicities such as hand-foot syndrome and transaminitis. These findings are consistent with landmark trials (e.g., IMbrave150 bleeding risks [13], REFLECT hepatotoxicity [32]) while extending evidence to triplet therapy contexts. Notably, the incidence of gastrointestinal hemorrhage in our bevacizumab cohort was higher than that reported in pivotal trials. This difference may stem from several study-specific factors. Patients in our cohort had advanced disease with frequent portal vein tumor thrombosis, suggesting a significant background of portal hypertension. Additionally, the fluorouracil component of FOLFOX-HAIC can cause mucositis, potentially acting synergistically with bevacizumab to increase gastrointestinal mucosal vulnerability. It should be noted that all hemorrhagic events occurred in patients who were considered appropriate candidates for bevacizumab therapy following thorough clinical evaluation, and most events were low-grad. However, in the context of portal hypertension, even low-grade bleeding warrants clinical attention, as it may signal a risk of more severe hemorrhage. This incidence rate should be regarded as a significant safety warning for this treatment regimen in HCC patients with portal vein tumor thrombosis. It is emphasized that in future clinical practice, for patients considered for bevacizumab-containing combination therapy who have risk factors for portal hypertension, baseline endoscopic screening and necessary preventive measures (such as ligation) are crucial and indispensable.

This study has several limitations. Its retrospective, single-center nature introduces inherent selection bias and limits generalizability. Clinically relevant baseline imbalances, particularly in the rates of extrahepatic metastasis and cirrhosis, were present between the groups. Although baseline characteristics were balanced, the modest sample size reduced statistical power and hindered comprehensive subgroup analyses, particularly in the heterogeneous TKIs group, where the influence of specific agents on toxicity could not be adequately assessed. Furthermore, the follow-up duration remains insufficient for mature OS analysis, and the emerging survival advantage in the bevacizumab group may become more pronounced with extended observation. These limitations highlight the need for future multicenter, prospective studies with larger cohorts to validate our findings.

## 5. Conclusions

This retrospective study suggests that the triplet therapy combining HAIC, ICIs, and anti-angiogenic therapy is a promising approach for advanced HCC, and the bevacizumab-based regimen was associated with better efficacy outcomes than TKI-based regimens. Both regimens demonstrated manageable, yet distinct, toxicity profiles, supporting their clinical viability with appropriate monitoring. This study observed a relatively high incidence of bleeding events with the bevacizumab-containing regimen. Therefore, we strongly recommend that the future clinical application of such regimens must be combined with mandatory baseline endoscopic risk assessment and appropriate secondary preventive measures to minimize patient bleeding risk. These preliminary findings, tempered by the study’s retrospective design, modest sample size, and heterogeneity within the TKIs group, highlight the potential of HAIC-based triplet therapy. Prospective, randomized trials with larger cohorts are warranted to validate these results, establish mature overall survival benefits, and optimize patient selection.

## Figures and Tables

**Table 1:** Comparison of patient baseline demographic and clinical characteristics between two groups.

Characteristics	Bevacizumab Group (*n* = 31)	TKIs Group (*n* = 34)	*p*-Value
Sex, No. (%)			>0.99
Male	29 (93.5)	32 (94.1)	
Female	2 (6.5)	2 (5.6)	
Age, No. (%), y			0.915
<55	15 (48.4)	16 (47.1)	
≥55	16 (51.6)	18 (52.9)	
Comorbidities, No. (%)			0.180
Absence	20 (64.5)	27 (79.4)	
Presence	11 (35.5)	7 (20.6)	
Etiology, No. (%)			0.935
Hepatitis B	29 (93.5)	33 (97.1)	
Hepatitis C	2 (6.5)	1 (2.9)	
Cirrhosis, No. (%)			0.113
Absence	17 (54.8)	12 (35.3)	
Presence	14 (45.2)	22 (64.7)	
Child-Pugh class, No. (%)			0.435
A	23 (74.2)	22 (64.7)	
B	8 (25.8)	12 (35.3)	
ALBI grade, No. (%)			0.716
1	9 (29.0)	7 (20.6)	
2	20 (64.5)	25 (73.5)	
3	2 (6.5)	2 (5.9)	
ECOG PS, No. (%)			0.057
0	25 (80.6)	20 (58.8)	
1	6 (19.4)	14 (41.2)	
Maximum tumor size, No. (%), cm			0.432
<10	10 (32.3)	8 (23.5)	
≥10	21 (67.7)	26 (76.5)	
Tumor distribution, No. (%)			0.285
Unilobar	15 (48.4)	12 (35.3)	
Bilobar	16 (51.6)	22 (64.7)	
Tumor number, No. (%)			0.189
<3	14 (45.2)	10 (29.4)	
≥3	17 (54.8)	24 (70.6)	
IVCTT, No. (%)			>0.99
Absence	26 (83.9)	29 (85.3)	
Presence	5 (16.1)	5 (14.7)	
Portal invasion, No. (%)			0.282
Vp0	4 (12.9)	7 (20.6)	
Vp1	4 (12.9)	3 (8.8)	
Vp2	5 (16.1)	5 (14.7)	
Vp3	13 (41.9)	7 (20.6)	
Vp4	5 (16.1)	12 (35.3)	
Extrahepatic spread, No. (%)			0.056
Absence	21 (67.7)	15 (44.1)	
Presence	10 (32.3)	19 (55.9)	
Both with portal invasion and extrahepatic spread, No. (%)			0.191
Absence	23 (74.2)	20 (58.8)	
Presence	8 (25.8)	14 (41.2)	
AFP, No. (%), ng/mL			0.592
<400	12 (38.7)	11 (32.4)	
≥400	19 (61.3)	23 (67.6)	
BMI, mean ± SD	23.2 ± 3.3	23.2 ± 4.0	0.984
NLR, mean ± SD	3.3 ± 2.5	3.6 ± 2.0	0.693

ALBI, albumin–bilirubin grade; ECOG PS, Eastern Cooperative Oncology Group Performance Status; IVCTT, inferior vena cava tumor thrombosis; AFP, α-fetoprotein; BMI, body mass index; NLR, Neutrophil-to-Lymphocyte Ratio.

**Table 2:** Comparison of efficacy between two groups.

	RECIST 1.1		
	Bevacizumab Group (*n* = 31)	TKIs Group (*n* = 34)	*p* Value
The best overall response, No (%)			
CR	0 (0.0)	0 (0.0)	
PR	26 (83.9)	21 (61.8)	
SD	4 (12.9)	8 (23.5)	
PD	1 (3.2)	5 (14.7)	
ORR	26 (83.9)	21 (61.8)	0.047
DCR	30 (96.8)	29 (85.3)	0.243
TTR, median (95% CI), month	2.3 (1.6–3.0)	3.0 (1.9–4.0)	0.366
DOR, median (95% CI), month	7.9 (4.9–10.8)	5.3 (3.0–7.7)	0.008
PFS, median (95% CI), month	10.9 (8.5–13.4)	7.4 (5.3–9.5)	0.001
OS, median (95% CI), month	NR (NR–NR)	23.0 (18.1–27.8)	0.523
6-month OS rate, %	100.0	91.2	
12-month OS rate, %	83.9	73.5	
18-month OS rate, %	57.1	58.8	

CR, complete response; PR, partial response; SD, stable disease; PD, progressive disease; ORR, objective response rate; DCR, disease control rate; TTR, time to response; DOR, duration of response; PFS, progression-free survival; OS, overall survival; CI, confidence interval; NR, not reached.

**Table 3:** Univariate and multivariate analysis of progression-free survival.

	PFS (RECIST 1.1)					
	Univariable Analysis			Multivariable Analysis		
	HR	95% CI	*p* Value	HR	95% CI	*p* Value
Sex (male vs. female)	0.60	0.22–1.68	0.329			
Age (≥55 vs. <55)	0.76	0.44–1.30	0.317			
Comorbidities (presence vs. absence)	0.63	0.33–1.18	0.145			
Etiology (HBV vs. HCV)	1.55	0.38–6.38	0.542			
Cirrhosis (presence vs. absence)	1.46	0.84–2.53	0.178			
Child-Pugh class (B vs. A)	1.66	0.94–2.95	0.082			
ALBI grade (3 vs. 1–2)	2.18	0.77–6.15	0.142			
ECOG PS (1 vs. 0)	1.86	1.06–3.29	0.032	1.57	0.87–2.82	0.313
Tumor size (≥10 cm vs. <10 cm)	1.67	0.90–3.09	0.103			
Tumor number (≥3 vs. <3)	1.69	0.94–3.06	0.080			
Tumor distribution (bilobar vs. unilobar)	1.54	0.88–2.71	0.132			
HBsAg (positive vs. negative)	1.05	0.54–2.04	0.885			
IVCTT (presence vs. absence)	1.82	0.90–3.68	0.095			
Portal invasion (presence vs. absence)	0.67	0.33–1.37	0.271			
Extrahepatic spread (presence vs. absence)	2.35	1.36–4.07	0.002	2.20	0.98–4.94	0.055
Both with portal invasion and extrahepatic spread (presence vs. absence)	2.02	1.16–3.51	0.013	0.90	0.39–2.09	0.814
AFP (≥400 vs. <400)	1.13	0.64–2.00	0.676			
BMI	1.00	0.92–1.08	0.968			
NLR	1.05	0.96–1.16	0.293			
Treatment (bevacizumab vs. TKIs)	0.40	0.23–0.71	0.001	0.47	0.26–0.85	0.012

PFS, progression-free survival; ALBI, albumin–bilirubin grade; ECOG PS, Eastern Cooperative Oncology Group Performance Status; IVCTT, inferior vena cava tumor thrombosis; HBsAg, hepatitis B surface antigen; AFP, α-fetoprotein; BMI, body mass index; NLR, Neutrophil-to-Lymphocyte Ratio.

## Data Availability

Any further information can be requested from the corresponding author.

## Supplementary Materials

The following supporting information can be downloaded at https://www.mdpi.com/article/10.3390/cancers18020314/s1: Figure S1: Exploratory analysis of progression-free survival stratified by the specific anti-angiogenic agent; Table S1: Combination therapy regimen for all patients; Table S2: The administration of study treatment in two groups; Table S3: Treatment-related adverse events in two groups; Table S4: Summary of treatment adjustments due to TRAEs in two groups.
