# Supplementary material for: Efficacy and Safety of HAIC Combined with PD-(L)1 Inhibitors and Bevacizumab Versus HAIC with PD-(L)1 Inhibitors and TKIs in Advanced Hepatocellular Carcinoma: A Retrospective Cohort Study
Source: Cancers (Basel). 2026 Jan 20;18(2):314. doi: 10.3390/cancers18020314 (PMC12838740; doi:10.3390/cancers18020314)
Supplement: Supplementary file 1 [file cancers-18-00314-s001.zip › cancers-4042288-supplementary.pdf]

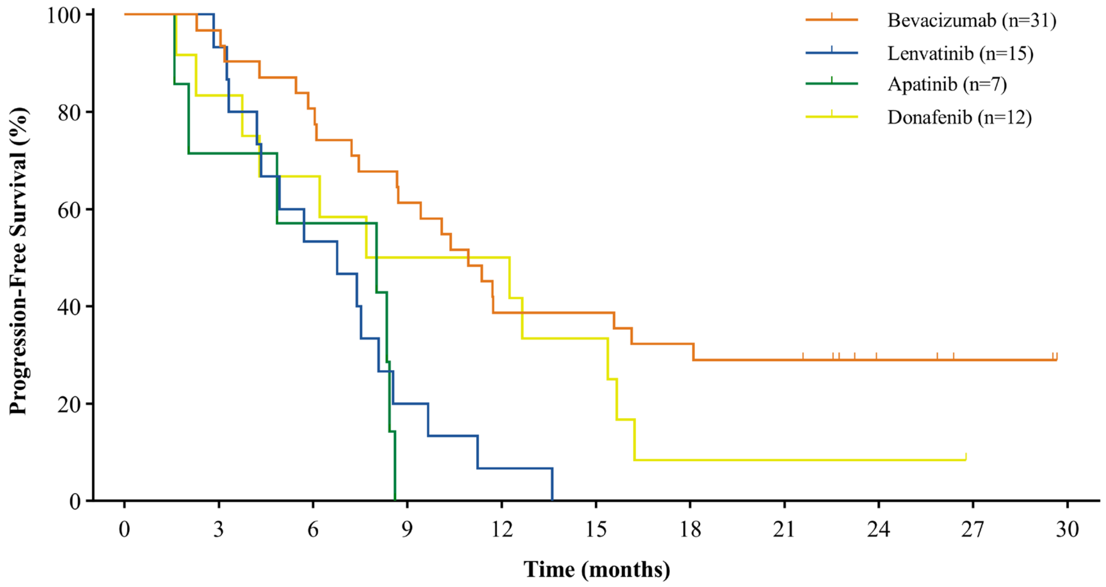

Group	Median (95% CI)	HR (95% CI)	P value			
			Bevacizumab	Lenvatinib	Apatinib	Donafenib
Bevacizumab	10.9 (8.5–13.4)			<0.001	0.001	0.231
Lenvatinib	6.8 (3.7–9.9)	5.115 (1.972–13.271)	<0.001		0.639	0.053
Apatinib	8.0 (0.0–16.1)	0.942 (0.322–2.753)	0.001	0.639		0.103
Donafenib	7.7 (0.0–17.9)	3.101 (1.331–7.221)	0.231	0.053	0.103	

Figure S1 Exploratory analysis of progression-free survival stratified by the specific anti-angiogenic agent.

Table S1 Combination therapy regimen for all patients

Combination therapy regimen, No (%)	Patients (n=65)
HAIC + Bevacizumab + Sintilimab	30 (46.2)
HAIC + Lenvatinib + Tislelizumab	8 (12.3)
HAIC + Apatinib + Camrelizumab	7 (10.8)
HAIC + Donafenib + Camrelizumab	5 (7.7)
HAIC + Lenvatinib + Sintilimab	5 (7.7)
HAIC + Donafenib + Triplimab	4 (6.2)
HAIC + Donafenib + Tislelizumab	3 (4.6)
HAIC + Bevacizumab + Atezolizumab	1 (1.5)
HAIC + Lenvatinib + Atezolizumab	1 (1.5)
HAIC + Lenvatinib + Camrelizumab	1 (1.5)

HAIC, hepatic arterial infusion chemotherapy

Table S2 The administration of study treatment in two groups

Study treatment	Bevacizumab group (n=31)	TKIs group (n=34)
PD-(L)1 inhibitor, No. (%)		
Sintilimab	30 (96.8)	5 (14.7)
Tislelizumab	0 (0.0)	11 (32.4)
Camrelizumab	0 (0.0)	13 (38.2)
Atezolizumab	1 (3.2)	1 (2.9)
Triplimab	0 (0.0)	4 (11.8)
Targeted drug, No. (%)		
Apatinib	-	7 (20.6)
Donafenib	-	12 (35.3)
Lenvatinib	-	15 (44.1)
Bevacizumab	31 (100.0)	-
Treatment management, median (range)		
HAIC cycle	6 (3–9)	5 (2–9)
ICIs cycle	11 (3–33)	7 (2–33)
Anti-angiogenic therapy cycle	11 (3–33)	6 (1–24)

HAIC, hepatic arterial infusion chemotherapy; ICIs, immune checkpoint inhibitors; PD-(L)1, programmed death-(ligand)1

Table S3 Treatment-related adverse events in two groups

Events, No. (%)	Any grade			Grade 1–2		Grade 3–4	
	Bev group (n=31)	TKIs group (n=34)	*P* value	Bev group (n=31)	TKIs group (n=34)	Bev group (n=31)	TKIs group (n=34)
Any TEAEs	31 (100.0)	34 (100.0)	1.000	31 (100.0)	34 (100.0)	21 (67.7)	25 (73.5)
LYMP count decreased	20 (64.5)	16 (47.1)	0.252	12 (38.7)	12 (35.3)	8 (25.8)	4 (11.8)
PLT count decreased	21 (67.7)	17 (50.0)	0.183	18 (58.1)	12 (35.3)	3 (9.7)	5 (14.7)
NE count decreased	17 (54.8)	13 (38.2)	0.281	12 (38.7)	11 (32.4)	5 (16.1)	2 (5.9)
Anemia	11 (35.5)	6 (17.6)	0.218	10 (32.3)	5 (14.7)	1 (3.2)	1 (2.9)
Decreased WBC count	11 (35.5)	8 (23.5)	0.693	10 (32.3)	7 (20.6)	1 (3.2)	1 (2.9)
AST increased	10 (32.3)	23 (67.6)	0.003	9 (29.0)	13 (38.2)	1 (3.2)	10 (29.4)
BILI increased	7 (22.6)	10 (29.4)	0.583	7 (22.6)	10 (29.4)	0 (0.0)	0 (0.0)
ALT increased	6 (19.4)	21 (61.8)	0.002	5 (16.1)	17 (50.0)	1 (3.2)	4 (11.8)
GGT increased	5 (16.1)	3 (8.8)	0.556	4 (12.9)	3 (8.8)	1 (3.2)	0 (0.0)
Blood LDH increased	5 (16.1)	2 (5.9)	0.244	5 (16.1)	2 (5.9)	0 (0.0)	0 (0.0)
AP increased	2 (6.5)	2 (5.9)	1.000	2 (6.5)	2 (5.9)	0 (0.0)	0 (0.0)
Hypoalbuminemia	15 (48.4)	17 (50.0)	1.000	14 (45.2)	15 (44.1)	1 (3.2)	2 (5.9)
Hypertension	18 (58.1)	18 (52.9)	0.938	14 (45.2)	15 (44.1)	4 (12.9)	3 (8.8)
Proteinuria	5 (16.1)	10 (29.4)	0.300	5 (16.1)	9 (26.5)	0 (0.0)	1 (2.9)
CTnI increased	1 (3.2)	0 (0.0)	0.477	1 (3.2)	0 (0.0)	0 (0.0)	0 (0.0)
Abdominal pain	16 (51.6)	18 (52.9)	0.289	12 (38.7)	17 (50.0)	4 (12.9)	1 (2.9)
Gastrointestinal hemorrhage	14 (45.2)	3 (8.8)	0.002	13 (41.9)	3 (8.8)	1 (3.2)	0 (0.0)
Gastric ulcer	7 (22.6)	1 (2.9)	0.040	4 (12.9)	1 (2.9)	3 (9.7)	0 (0.0)
Nausea	5 (16.1)	10 (29.4)	0.248	5 (16.1)	10 (29.4)	0 (0.0)	0 (0.0)
Diarrhea	4 (12.9)	9 (26.5)	0.351	4 (12.9)	7 (20.6)	0 (0.0)	2 (5.9)
Constipation	3 (9.7)	4 (11.8)	1.000	3 (9.7)	4 (11.8)	0 (0.0)	0 (0.0)
Vomiting	3 (9.7)	3 (8.8)	1.000	3 (9.7)	3 (8.8)	0 (0.0)	0 (0.0)
Hand-foot syndrome	0 (0.0)	7 (20.6)	0.018	0 (0.0)	5 (14.7)	0 (0.0)	2 (5.9)
RCCEP	0 (0.0)	4 (11.8)	0.115	0 (0.0)	4 (11.8)	0 (0.0)	0 (0.0)
Rash	2 (6.5)	5 (14.7)	0.674	2 (6.5)	4 (11.8)	0 (0.0)	1 (2.9)
Hyperuricemia	2 (6.5)	3 (8.8)	1.000	2 (6.5)	3 (8.8)	0 (0.0)	0 (0.0)
TSH increased	2 (6.5)	2 (5.9)	1.000	2 (6.5)	2 (5.9)	0 (0.0)	0 (0.0)
Hypothyroidism	1 (3.2)	1 (2.9)	1.000	1 (3.2)	1 (2.9)	0 (0.0)	0 (0.0)

CPK increased	1 (3.2)	3 (8.8)	0.615	1 (3.2)	3 (8.8)	0 (0.0)	0 (0.0)
Weight loss	13 (41.9)	15 (44.1)	1.000	13 (41.9)	15 (44.1)	0 (0.0)	0 (0.0)
Fever	10 (32.3)	14 (41.2)	0.608	10 (32.3)	14 (41.2)	0 (0.0)	0 (0.0)
Fatigue	9 (29.0)	11 (32.4)	0.795	9 (29.0)	11 (32.4)	0 (0.0)	0 (0.0)
Ascites	2 (6.5)	2 (5.9)	0.795	2 (6.5)	1 (2.9)	0 (0.0)	1 (2.9)
Lung infection	2 (6.5)	1 (2.9)	0.735	1 (3.2)	1 (2.9)	1 (3.2)	0 (0.0)
Abdominal infection	0 (0.0)	1 (2.9)	1.000	0 (0.0)	0 (0.0)	0 (0.0)	1 (2.9)
Anaphylaxis	1 (3.2)	0 (0.0)	0.477	1 (3.2)	0 (0.0)	0 (0.0)	0 (0.0)

LYMP, lymphocyte; PLT, platelet; NE, neutrophil; WBC, white blood cell; AST, aspartate aminotransferase; ALT, alanine aminotransferase; LDH, lactate dehydrogenase; GGT, gamma–glutamyl transferase; TSH, thyroid stimulating hormone; AP, alkaline phosphatase; CTnI, Cardiac troponin I; BILI, bilirubin; CPK, creatine phosphokinase; RCCEP, reactive cutaneous capillary endothelial proliferation

Table S4 Summary of treatment adjustments due to TRAEs in two groups

Events, No. (%)	HIB group (n=31)	HIT group (n=34)
TRAEs leading to dose reduction	0 (0.0)	9 (26.5)
HAIC	0 (0.0)	0 (0.0)
ICIs	0 (0.0)	0 (0.0)
Anti-angiogenic therapy	0 (0.0)	9 (26.5)
TRAEs leading to dose interruption	9 (29.0)	1 (2.9)
HAIC	1 (3.2)	0 (0.0)
ICIs	4 (12.9)	0 (0.0)
Anti-angiogenic therapy	7 (22.6)	1 (2.9)
TRAEs leading to treatment discontinuation	1 (3.2)	1 (2.9)
HAIC	0 (0.0)	0 (0.0)
ICIs	1 (3.2)	0 (0.0)
Anti-angiogenic therapy	1 (3.2)	1 (2.9)

TRAEs, treatment-related adverse events; HAIC, hepatic arterial infusion chemotherapy; ICIs, immune checkpoint inhibitors.
